# Primary non-Hodgkin lymphoma in the muscle of left lower extremity: a case report and literature review

**DOI:** 10.3389/fonc.2025.1679393

**Published:** 2025-11-18

**Authors:** Haixia Du, Yongting Zhang, Hong Zhou, Xiaoguang Huo, Mengying Xu, Wenzhe Xu

**Affiliations:** 1 Zibo Hospital of Traditional Chinese, Medicine Functional Inspection Unit, Zibo, Shandong, China; 2 Department of Ultrasound, Zibo Central Hospital, Zibo, Shandong, China

**Keywords:** lower limb muscle, ultrasound diagnosis, magnetic resonance imaging diagnosis, pathological diagnosis, non-Hodgkin lymphoma

## Abstract

Primary non-Hodgkin lymphoma (NHL) of the left lower extremity muscle is a rare extra-nodal lymphoma, accounting for less than 1% of all extra-nodal NHL cases. We report a rare case of intramuscular NHL of the left lower extremity and discuss its clinical features, diagnostic challenges, and treatment strategies with reference to the literature. The patient, an adult man, was treated for sudden swelling and pain in the left lower limb. Ultrasound examination revealed hypoechoic myometrium (hematoma considered), and ultrasound-guided puncture biopsy confirmed “primary CD5+ diffuse large B-cell lymphoma (leg type)”. The patient received six cycles of chemotherapy, and his symptoms were significantly relieved, but an early local recurrence occurred. The clinical manifestations and imaging features of primary NHL in skeletal muscle lacked specificity, and the diagnosis depended on pathology and molecular analysis. Its pathogenesis may be related to direct invasion, hematogenous metastasis, or primary muscle lesions. CD5+ diffuse large B-cell lymphoma (DLBCL) is aggressive, and Rituximab-Cyclophosphamide Hydroxydaunorubicin Vincristine Prednisone (R-CHOP) combined with radiotherapy is recommended, but prognosis is affected by age, Lactate Dehydrogenase (LDH) levels, and molecular characteristics such as TP53 mutations. Primary non-Hodgkin lymphoma in the muscle of the left lower limb is very rare. It is difficult to diagnose it by imaging examination alone. It needs to be considered comprehensively by combining various examination methods. Pathology is the gold standard for diagnosis. Radiotherapy and chemotherapy are the first choice for treatment. It is very important to formulate a reasonable treatment plan according to the results of pathology and molecular analysis.

## 1 Introduction

Non-Hodgkin lymphoma (NHL) is a malignant tumor originating in the lymphatic system and characterized by high heterogeneity. Although NHL most often involves lymph nodes, approximately 40% of cases may present as extra-nodal involvement (1, 2). Primary lymphoma of skeletal muscle is extremely rare, accounting for less than 1% of all extra-nodal NHL cases.

In clinical practice, the diagnosis of skeletal muscle lymphoma often faces many challenges. First, its clinical manifestations lack specificity, often presenting as local masses or pain, and are easily confused with other soft tissue tumors or inflammatory diseases (3, 4). Second, imaging features do not have definite diagnostic value, and histopathological examination is required to confirm the diagnosis. Furthermore, the pathogenesis of skeletal muscle lymphoma is not fully understood and may be related to multiple pathways, such as direct invasion, hematogenous metastasis, or primary muscle pathology (5, 6).

The lower extremities, especially the thighs, are one of the most common sites of skeletal muscle lymphoma (7). Because of the complex anatomical structure of this region and the variety of clinical manifestations, early diagnosis is more difficult. This article reports a case of left lower limb intramuscular non-Hodgkin lymphoma and discusses the related literature in order to improve the understanding and diagnosis of this rare disease.

## 2 Case report

**Figure 1:**
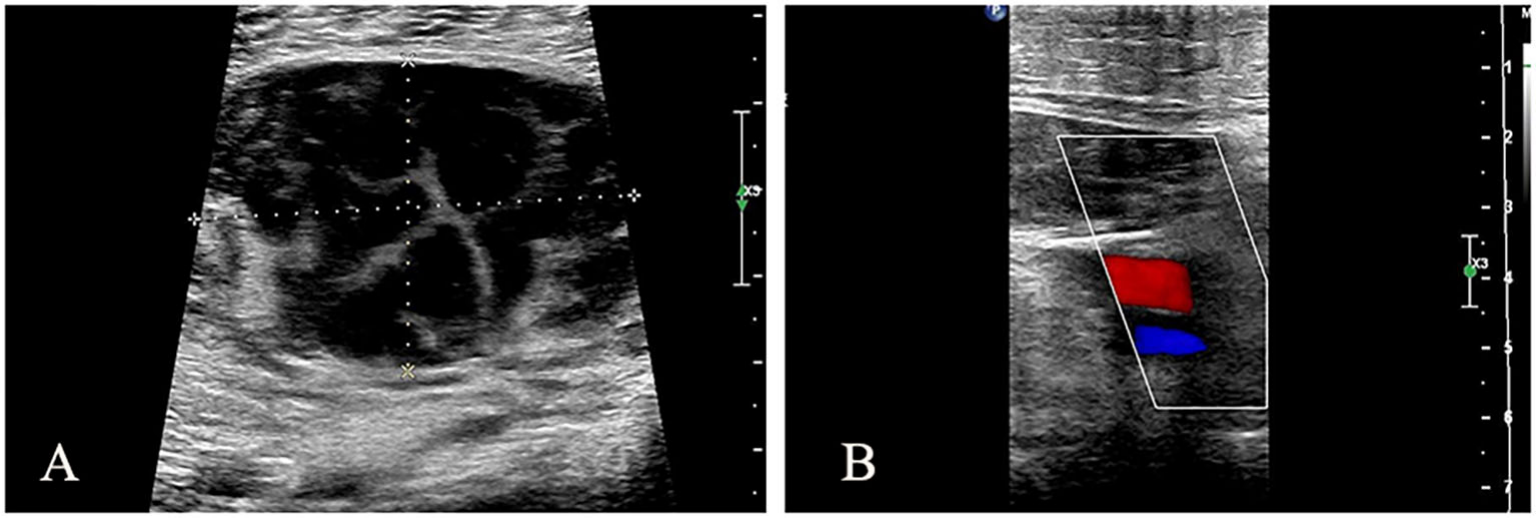
Ultrasound Doppler image of left lower extremity. **(A)** Hypoechoic area in left thigh muscle layer, approximately 51 × 36 mm, with clear boundary and uneven internal echo. **(B)** CDFI: No obvious blood flow signal was observed.

**Figure 2:**
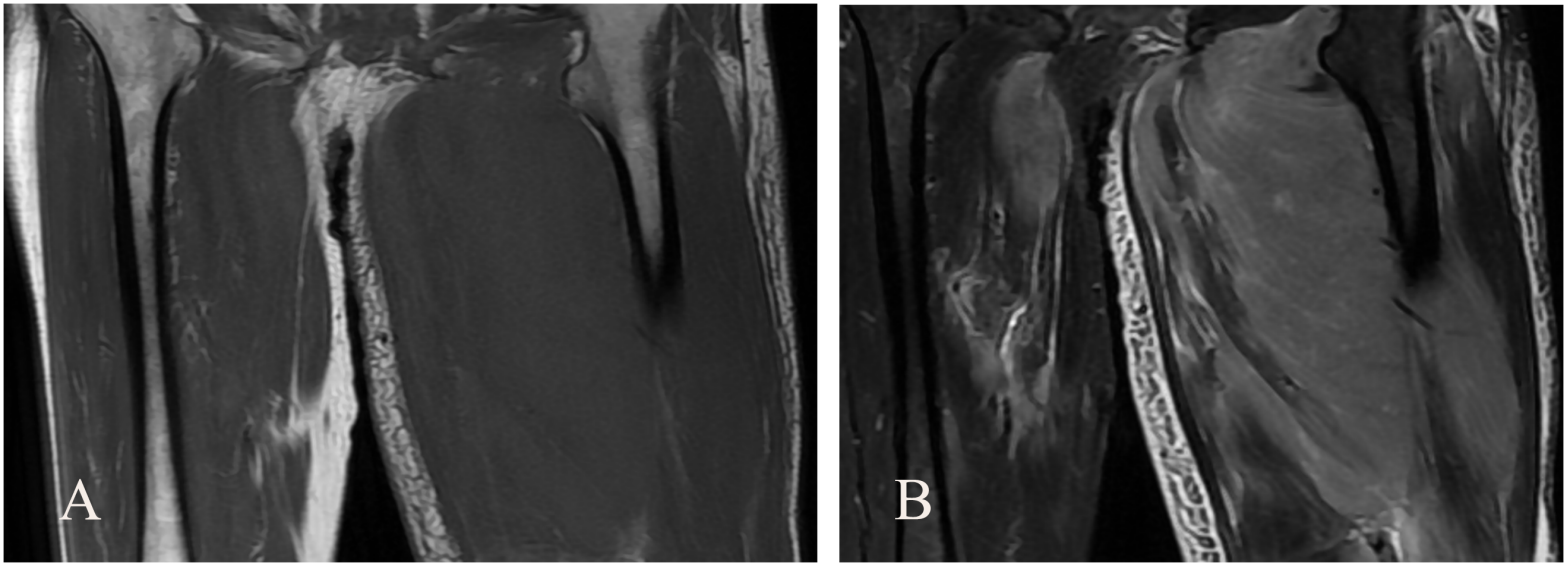
Coronal MRI images of lower extremities. **(A)** T1-weighted imaging (T1WI) sequence shows patchy long T1 abnormal low signal intensity in the middle and upper muscular layer of left thigh. **(B)** T2-weighted imaging (T2WI) sequence showed patchy long T2 abnormal high signal intensity in the middle and upper muscular layer of left thigh.

**Figure 3:**
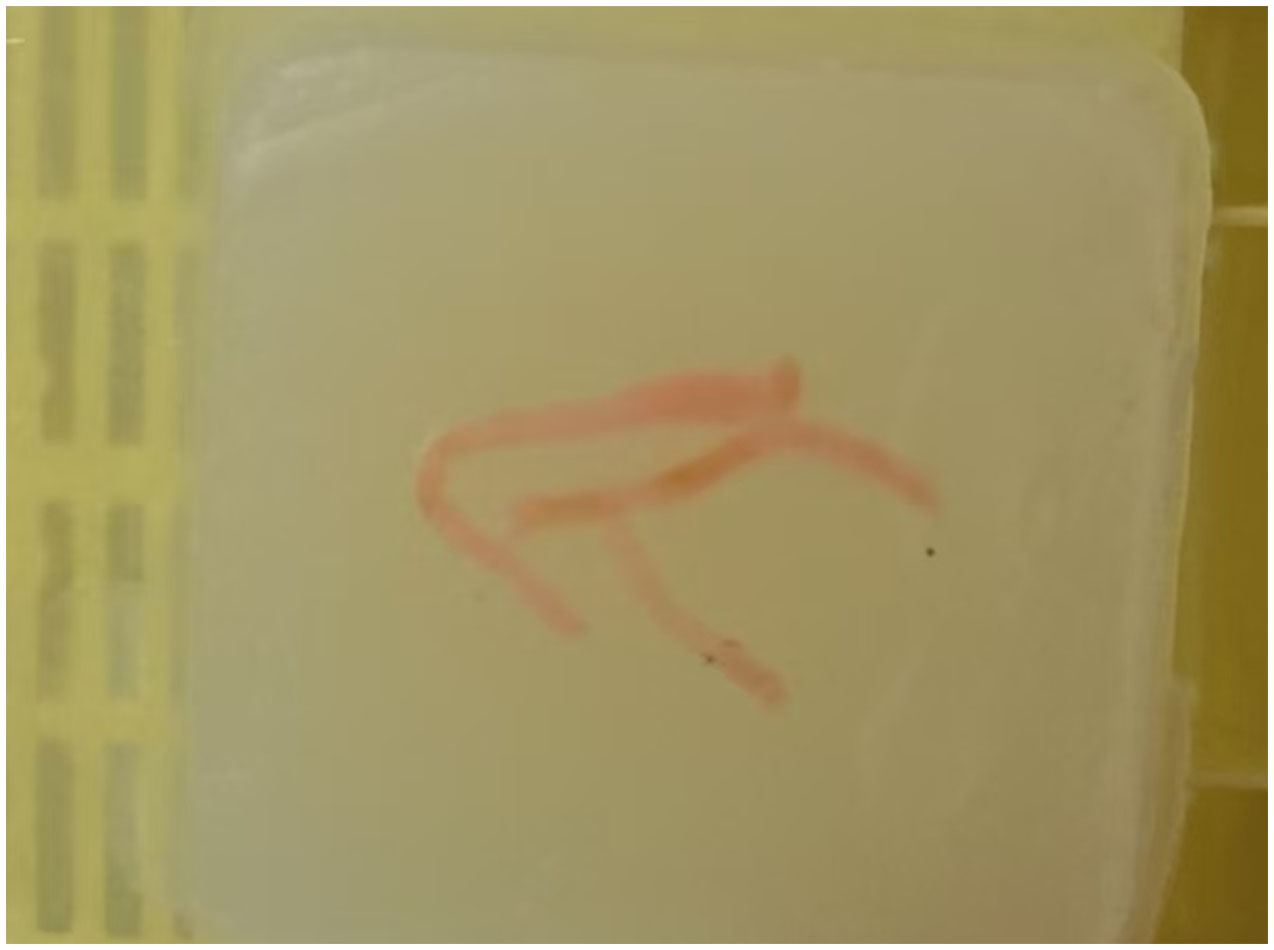
Post-operative specimen images. Three bands of paraffin-fixed, H&E-stained specimens are visible.

**Figure 4:**
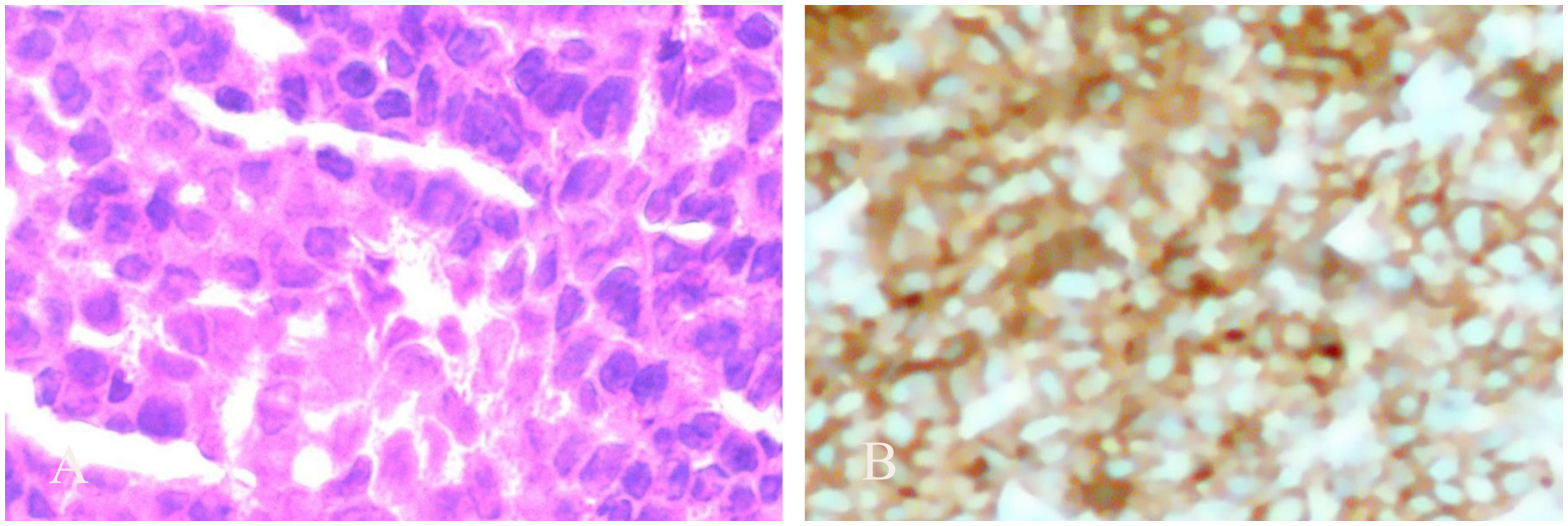
Pathological and immunohistochemical images. **(A)** Pathological picture shows diffuse hyperplasia of atypical medium-large lymphocytes (10 × 10). **(B)** Immunohistochemical CD5+ plot (10 × 10 times).

A 72-year-old male patient visited our hospital. Ten days ago, the patient experienced sudden swelling and pain in the left lower limb without an obvious cause, which was aggravated after standing and walking. There was no fever, headache, dizziness, chest distress, suffocation, chest pain, hemoptysis, cold skin, skin color flushing, or skin blisters. The symptoms gradually aggravated. He came to the hospital without special treatment. Color Doppler examination revealed a low-echo lesion in the left thigh muscle layer, measuring approximately 51 × 36 mm, with a clear boundary and heterogeneous echotexture. Color Doppler Flow Imaging (CDFI) indicated no obvious blood flow signal, and ultrasound diagnosis indicated low echo in the left thigh muscle layer (hematoma considered) (**Figure 1**). MRI plain scan showed no abnormality in the left femur shape; a patchy long T1 and long T2 signal was observed in the soft tissue around the middle and upper part of the left thigh, and the lesion was relatively large and diffuse in nature, with indistinct boundaries and an irregular shape. The internal signal was uniform, and no obvious signs of liquefaction or necrosis were observed. The diagnosis was left thigh muscle thickening with signal changes (**Figure 2**). Ultrasound-guided biopsy was performed for a definite diagnosis. Pathology indicated that three small strips of the left lower limb muscle biopsy tissue (**Figure 3**) showed atypical medium-large lymphocyte diffuse hyperplasia. Combined with immunohistochemistry, non-Hodgkin lymphoma was considered as “primary CD5+ diffuse large B-cell lymphoma” and “leg type” with focal necrosis. Immunohistochemistry indicated the following: CD20 (+); CD79a (+); CD5 (+); Bcl-2 (+)S accounted for 95%; Pax-5 (+); Vimentin (+); CD3, scattered (+); Mum-1, focal (+); Cyclin D1, SOX11, Bcl-6, CD10, CD23, CD30, ALK, CD38, CD138, S-100, MyoD1, CD99, CKAE1/AE3 (−); c-myc (++)S accounted for 90%; P53 (+)S accounted for 2%; Ki-67 (+)S (**Figure 4**). After a definite diagnosis, the patient underwent six cycles of chemotherapy with the R-CHOP regimen, with good effect. In this case, the patient achieved a partial response (PR) with chemotherapy. At present, the patient is being followed up continuously.

## 3 Discussion

Primary NHL of the left lower extremity muscle is a rare type of extra-nodal lymphoma, accounting for less than 1% (8) of all extra-nodal NHL cases reported in the literature. This case presented with sudden pain and edema in the muscles of the left lower extremity, consistent with typical symptoms described in the literature. It is worth noting that muscle lymphoma is often misdiagnosed as soft tissue sarcoma or inflammatory myopathy, with a misdiagnosis rate of 0.11% (9), suggesting that lymphoma should be considered in the differential diagnosis of soft tissue masses (10); imaging features are not specific, and the final diagnosis still depends on histopathology (11).

There are three possible mechanisms for the development of muscle lymphoma: direct invasion of adjacent bone/lymph nodes, hematogenous metastasis, or primary muscle lymphoma (12). This case met the definition of primary muscle lymphoma (8) by excluding other lesions through systemic staging. Molecular pathological analysis revealed that this case was primary CD5+ diffuse large B-cell lymphoma “leg type” with focal necrosis. CD5+ diffuse large B-cell lymphoma (DLBCL) is a rare subtype with aggressive clinical behavior characterized by a high frequency of MYD88^L265P^ mutations and CD79B mutations associated with persistent activation of the B-cell receptor (BCR) signaling pathway (13). Furthermore, CD5 expression is associated with abnormal activation of the NF-κB pathway and may partially overlap with molecular features of activated B-cell (ABC) subtypes (14), suggesting that different subtypes may have distinct clinical behaviors.

The R-CHOP regimen combined with radiotherapy is recommended for primary muscular DLBCL (15–17). This case achieved a partial response with chemotherapy, but the risk of local recurrence should be considered. The literature shows that the 5-year survival rate of muscle lymphoma is approximately 65%. Age > 60 years and elevated LDH are poor prognostic factors (18). It is particularly noteworthy that this case has local recurrence in the left lower limb after treatment, similar to the muscle recurrence pattern reported in the literature (9), suggesting that this site may have special tumor microenvironment resistance.

Compared with the previously reported DLBCL in the right lower limb muscle of an 82-year-old male patient (19, 20), 70-year-old male thigh case (7), and left thigh recurrence case (12), the particularity of this case lies in the following: 1) the lesion is limited to the left lower limb thigh muscle group, and the anatomical location is more rare; 2) early local recurrence occurs immediately after the first treatment, suggesting that there may be unique tumor biological characteristics; and 3) TP53 mutation was found by second-generation sequencing, which has not been reported in the literature in muscle lymphoma and may provide direction for future targeted therapy. DLBCL subtypes are more sensitive to chemotherapy than mantle cell lymphoma involving the left thigh in case 9, but long-term control remains challenging (**Table 1**).

**Table 1:** Cases of primary non-Hodgkin lymphoma reported.

First author, year	Number	Histopathological diagnosis	Therapeutic approach	References
Shuxi Gao, 2021	1	Positive for CD20, CD19, Vimentin, Bcl-2, Bcl-6, and MUM-1	R-CHOP	(7)
Nikolaos Spetsieris, 2018	1	Positive for CD30, with a Ki-67 proliferation rate of 90%	R-CHOP	(12)
Sha Ma, MD, 2022	207	Positive for CD5	R-CHOP/CHOP	(13)
Kana Miyazaki, 2020	47	Positive for CD5	Dose-Adjusted Etoposide Prednisone Vincristine Cyclophosphamide Doxorubicin-Rituximab/High-Dose Methotrexate (DA-EPOCH-R/HD-MTX)	(14)
Daniel O Persky, 2020	158	Positive for CD20	R-CHOP	(16)

## 4 Conclusion

Muscular primary NHL is a rare malignant tumor that is prone to recurrence after operation and lacks typical imaging findings. At present, there are no generally accepted guidelines for preoperative diagnosis and treatment. Through the study of the chemotherapy regimen of this case and review of related literature, we hope to help clinicians have a deeper understanding of this disease and provide a reference for preoperative evaluation and formulation of a reasonable treatment regimen.

## Data Availability

The original contributions presented in the study are included in the article/supplementary material. Further inquiries can be directed to the corresponding author.

## References

[1] Mansour S Abdul Rahman SA Mansour M Afif A Hasan R Abdullah . Relapse of diffuse large B-cell lymphoma as painless masses in the abdominal wall muscles: A rare case report. Cancer Rep (Hoboken NJ). (2025) 8:e70114. doi: 10.1002/cnr2.70114, PMID: 39763214 PMC11705403

[2] Yang H Xun Y Ke C Tateishi K You H . Extranodal lymphoma: pathogenesis, diagnosis and treatment. Mol Biomed. (2023) 4:29. doi: 10.1186/s43556-023-00141-3, PMID: 37718386 PMC10505605

[3] Celayir A Afacan MY Sevgil B Özsahin MK Botanlioglu H . A rare case of non-hodgkin lymphoma with ankle involvement: diagnostic and therapeutic challenges. Cureus. (2025) 17:e77987. doi: 10.7759/cureus.77987, PMID: 40013207 PMC11859464

[4] Odate T Satomi K Kubo T Matsushita Y Ueno T Kurose A . Inflammatory rhabdomyoblastic tumor: clinicopathologic and molecular analysis of 13 cases. Modern Pathol. (2024) 37:100359. doi: 10.1016/j.modpat.2023.100359, PMID: 37871654

[5] Binici DNR Karaman A Timur O Tasar PNT Sanibas AV . Primary skeletal muscle lymphoma: A case report. Mol Clin Oncol. (2018) 8:80–2. doi: 10.3892/mco.2017.1483, PMID: 29387400 PMC5768100

[6] Ran P Li C Lv J Liang X Dong A . 18 F-FAPI-42 versus 18 F-FDG PET/MRI in a case of primary peripheral T-cell lymphoma of the skeletal muscles. Clin Nucl Med. (2024) 49:757–60. doi: 10.1097/RLU.0000000000005311, PMID: 38861415

[7] Gao S Shu H Yang H . Imaging features of skeletal muscle lymphoma: a case report and literature review. BMC Med Imaging. (2021) 21:136. doi: 10.1186/s12880-021-00667-4, PMID: 34565344 PMC8474738

[8] Ankita Ahuja S Malik S Zaheer S . Primary extranodal diffuse large b-cell lymphoma of the calf muscle mimicking a carcinoma: A rare case report. Int J Surg Case Rep. (2024) 123:110298. doi: 10.1016/j.ijscr.2024.110298, PMID: 39293218 PMC11421246

[9] Christy J Kandah E Kesari K Peram V . Primary soft tissue sarcoma: stage IV extranodal T-cell non-Hodgkin's lymphoma. BMJ Case Rep. (2021) 14. doi: 10.1136/bcr-2021-243243, PMID: 34326112 PMC8323354

[10] Granadas J Baptista M Ferreira S . Lymphoma presenting as a soft tissue mass. J Belgian Soc Radiol. (2022) 106:94. doi: 10.5334/jbsr.2893, PMID: 36310676 PMC9563229

[11] Duong LT Rollin M Bidault F Lazarovici J Ferré FC . Orofacial intramuscular lymphoma: first presentation. Ear Nose Throat J. (2022) 104:1455613221101940. doi: 10.1177/01455613221101940, PMID: 35794792

[12] Spetsieris N Giannakopoulou N Variami E Zervakis K Rougala N Garefalakis G . Isolated skeletal muscle recurrence of an originally nodal diffuse large B cell lymphoma: A case report and review of the literature. Medicine. (2018) 97:e9608. doi: 10.1097/MD.0000000000009608, PMID: 29504987 PMC5779756

[13] Ma S Zhang B Lu T Li D Li T Shen Z . Value of the prognostic nutritional index (PNI) in patients with newly diagnosed, CD5-positive diffuse large B-cell lymphoma: A multicenter retrospective study of the Huaihai Lymphoma Working Group. Cancer. (2022) 128:3487–94. doi: 10.1002/cncr.34405, PMID: 35932292

[14] Miyazaki K Asano N Yamada T Miyawaki K Sakai R Igarashi T . DA-EPOCH-R combined with high-dose methotrexate in patients with newly diagnosed stage II-IV CD5-positive diffuse large B-cell lymphoma: a single-arm, open-label, phase II study. Haematologica. (2020) 105:2308–15. doi: 10.3324/haematol.2019.231076, PMID: 33054055 PMC7556618

[15] Batgi H Merdin A Dal MS Kızıl Çakar M Yıldız J Basçı S . The effect of gemcitabine, dexamethasone, and cisplatin chemotherapy in relapsed/refractory NHL and HL patients: A single center experience. J Oncol Pharm Pract. (2020) 26:1857–63. doi: 10.1177/1078155220905654, PMID: 32098553

[16] Persky DO Li H Stephens DM Park SI Bartlett NL Swinnen LJ . Positron emission tomography-directed therapy for patients with limited-stage diffuse large B-cell lymphoma: results of intergroup national clinical trials network study S1001. J Clin Oncol. (2020) 38:3003–11. doi: 10.1200/JCO.20.00999, PMID: 32658627 PMC7479758

[17] Zhang XY Collins GP Cutter DJ Eyre TA . Limited-stage diffuse large B-cell lymphoma: current management and challenges. Br J Haematol. (2021) 194:508–17. doi: 10.1111/bjh.17359, PMID: 33618434

[18] Lee CH Jeon SY Yhim HY Kwak JY . Disseminated soft tissue diffuse large B-cell lymphoma involving multiple abdominal wall muscles: A case report. World J Clin cases. (2021) 9:8557–62. doi: 10.12998/wjcc.v9.i28.8557, PMID: 34754868 PMC8554429

[19] Li C Hu X . Primary extranodal nasal-type natural killer/T-cell lymphoma of lower limb muscles on 18 F-FDG PET/CT. Clin Nucl Med. (2024) 49:e45–6. doi: 10.1097/RLU.0000000000004959, PMID: 37976527

[20] Hatem J Bogusz AM . An unusual case of extranodal diffuse large B-cell lymphoma infiltrating skeletal muscle: A case report and fi 2review of the literature. Case Rep Pathol. (2016) 2016:9104839. doi: 10.1155/2016/9104839, PMID: 27247818 PMC4877472

